# Responses of Processed Electroencephalographic Indices to Ascending and Descending Simulated Remimazolam Effect-Site Concentrations: A Prospective Exploratory Interventional Study

**DOI:** 10.3390/jcm15207905

**Published:** 2026-10-13

**Authors:** Sou-Hyun Lee, Hyungjun Kwon, Seong Wook Hong, Hyun-Su Ri, Jung A Lim, Chanhyo Choi, Tae Hoon Kang, Hoon Jung, Kyung-Hwa Kwak

**Affiliations:** 1 Department of Anesthesiology and Pain Medicine, Kyungpook National University Hospital, Kyungpook National University School of Medicine, Daegu 41944, Republic of Korea; 2 Department of Surgery, Kyungpook National University Hospital, Kyungpook National University School of Medicine, Daegu 41944, Republic of Korea; 3 Department of Anesthesiology and Pain Medicine, Kyungpook National University Chilgok Hospital, Kyungpook National University School of Medicine, Daegu 41404, Republic of Korea

**Keywords:** remimazolam, electroencephalography, consciousness monitors

## Abstract

**Background/Objectives**: Temporal characteristics of concentration–response loops for processed electroencephalographic indices during changes in remimazolam effect-site concentration (*Ce*) remain unclear. Therefore, this prospective, exploratory interventional study aims to compare the magnitude of concentration–response loops between the ascending and descending phases of simulated remimazolam *Ce* for the bispectral index (BIS) and patient state index (PSI). **Methods**: Twenty patients were administered remimazolam at a constant infusion rate of 3.5 mg/kg/h for 5.5 min, followed by infusion discontinuation. BIS and PSI values were continuously recorded from initiation of remimazolam infusion until 16 min, and remimazolam *Ce* was simulated using the Schüttler–Eisenried pharmacokinetic model. The area between the ascending and descending concentration–response curves (ABC) was calculated for each index within the overlapping *Ce* range. ABC was compared between BIS and PSI using a paired analysis, and a patient-level bootstrap was used to estimate the 95% confidence interval (CI) for the paired difference. **Results:** All 20 patients were included in the final analysis. The mean paired difference in ABC (BIS − PSI) was −6.254 (95% CI: −7.945 to −4.563), with a bootstrap median difference of −6.241 (95% CI: −8.752 to −4.133). The gap between the ascending and descending concentration–response curves was greater for PSI than for BIS. **Conclusions**: BIS and PSI showed different concentration–response patterns during changes in simulated remimazolam *Ce*, with PSI showing greater separation between the ascending and descending phases. These exploratory findings do not establish direct pharmacodynamic hysteresis or indicate superiority of BIS for assessing hypnotic state.

## 1. Introduction

Traditionally, continuous infusion of anesthetic agents for intravenous anesthesia is performed using target-controlled infusion (TCI). TCI uses a pump or computer-controlled system that automatically adjusts the infusion rate to maintain a predefined target concentration [1]. TCI controls drug effect by directly regulating the effect-site concentration (*Ce*) [2].

Effect-site TCI requires pharmacokinetic (PK) analysis to estimate the plasma concentration, followed by pharmacodynamic (PD) analysis to characterize the relationship between plasma concentration and drug effects, such as the patient state index (PSI) and bispectral index (BIS). Since the concentration–effect relationship based on plasma concentration often exhibits hysteresis owing to the time delay between plasma and effect sites, PD modeling is commonly used to estimate the plasma-to-effect-site equilibration rate constant (Ke0) and derive *Ce* [3]. Ce derived from the estimated Ke0 substantially reduces this hysteresis, enabling a more direct relationship between *Ce* and the PD effect.

Remimazolam is suitable for TCI due to its short context-sensitive half-life of ~7 min, even after a 4 h infusion [4]. Similarly to other benzodiazepines, PSI and BIS values typically remain above 50 and 60, respectively, despite loss of consciousness after remimazolam administration [5,6]. These relatively high index values are potentially attributable to the increased relative beta power after benzodiazepine administration [7].

To address this limitation, Schüttler et al. [4] developed a population PK/PD model using the Modified Observer’s Assessment of Alertness and Sedation (MOAA/S) scale as the effect variable. However, in clinical practice, effect-site TCI is commonly titrated using processed electroencephalogram (EEG) with BIS or PSI.

Although the associations of BIS and PSI have been examined with clinical sedation and remimazolam *Ce* during the ascending phase of drug administration [8,9], evidence regarding the behavior of BIS and PSI at ascending and descending phases remains limited. Therefore, this study aims to compare the divergence between the ascending and descending concentration–response curves of BIS and PSI according to the simulated remimazolam *Ce*. *Ce* was simulated using the Schüttler–Eisenried model, as in previous remimazolam studies [9,10,11].

## 2. Materials and Methods

### 2.1. Study Design and Participants

This prospective, single-center, exploratory interventional study was approved by the Institutional Review Board of Kyungpook National University Hospital (approval number: 2026-02-013; 2 March 2026). Before patient enrollment, the trial was registered with the Korean Clinical Research Information Service (https://cris.nih.go.kr/cris/search/detailSearch.do/35088; KCT0011686; principal investigator: Kyung-Hwa Kwak; registration date: 5 March 2026). The study was initially classified as observational in the registry; this classification was corrected to interventional on 2 October 2026, to reflect the prospectively assigned remimazolam dosing and observation protocol.

The study was conducted at the Kyungpook National University Hospital from 12 March to July 2026. All participants provided written informed consent 1 day before surgery. This study was reported following the Transparent Reporting of Evaluations with Nonrandomized Designs (TREND) guidelines.

Eligible participants were adults aged ≥ 19 years with an American Society of Anesthesiologists physical status (ASA-PS) of I or II, scheduled to undergo elective laparoscopic cholecystectomy. Exclusion criteria included a history of benzodiazepine or opioid allergy, alcohol dependence, neurological or psychiatric disorders, pregnancy, preexisting liver cirrhosis, or end-stage renal disease.

### 2.2. Anesthetic Induction Protocol

Standard physiological monitoring was instituted, including pulse oximetry, three-lead electrocardiography, noninvasive blood pressure measurement and capnography. Anesthetic depth was monitored concurrently using two processed EEG devices: PSI (SedLine^®^, Masimo Corporation, Irvine, CA, USA) and BIS (BIS^®^, Medtronic plc, Dublin, Ireland). Before sensor application, the forehead was cleansed with an alcohol swab; the PSI and BIS sensors were then simultaneously applied according to the instructions of the manufacturer. Throughout the observation period, patients were instructed to maintain eye closure to minimize electromyographic interference.

Patients underwent preoxygenation with 100% oxygen at 10 L/min for 5 min. Remimazolam (50 mg) was diluted in 20 mL of normal saline and administered using a syringe pump (Pilot Anesthesia 2, Fresenius Vial, Brézins, France) connected to a personal computer via an RS-232C cable. The pump was controlled using Asan Pump software (version 2.1.3, Bionet Co., Ltd., Seoul, Republic of Korea) installed on the same computer. Remimazolam was administered at a fixed rate of 3.5 mg/kg/h for 5.5 min, followed by infusion stoppage. No additional anesthetic agents were administered during the subsequent observation period, which continued until 16 min after initiation of the remimazolam infusion. Apnea was defined as the absence of an end-tidal CO_2_ waveform for >10 s. Apnea during this period was managed with assisted ventilation using a face mask.

**Figure 1:**
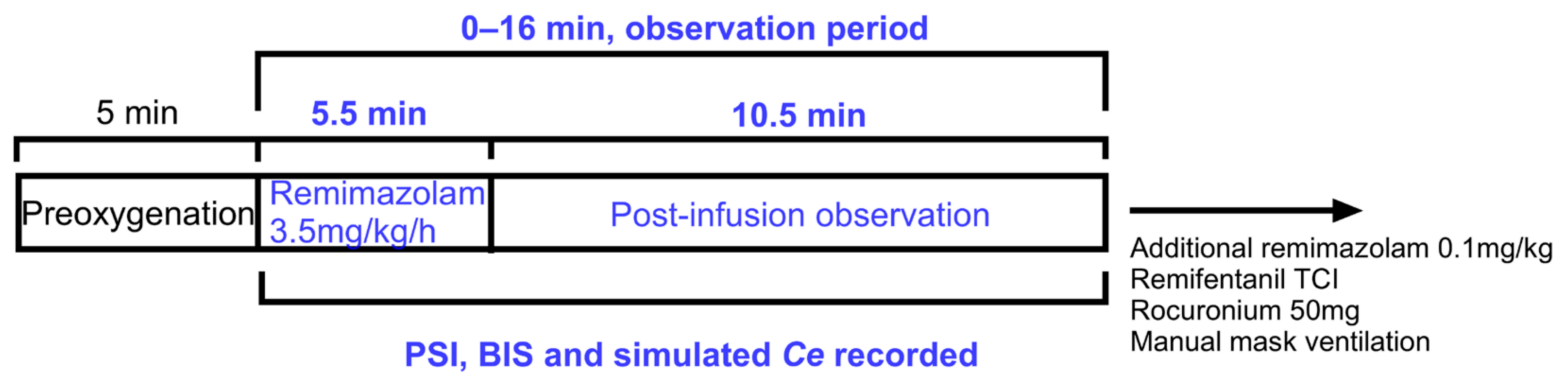
Timeline of anesthetic induction and data recording. PSI, BIS, and simulated remimazolam Ce were recorded from the initiation of the remimazolam infusion until 16 min thereafter. Abbreviations: PSI, patient state index; BIS, bispectral index; TCI, target-controlled infusion; *Ce*, effect-site concentration.

After the observation period, anesthesia was induced with an additional remimazolam bolus (0.1 mg/kg), rocuronium (50 mg), and remifentanil administered via TCI, followed by manual ventilation using a bag-valve-mask anesthesia system (Figure 1).

### 2.3. Outcomes and Definitions

BIS and PSI measurements were continuously captured at 2 s intervals using VitalRecorder software (version 1.17.1; VitalDB, Department of Anesthesiology and Pain Medicine, Seoul National University Hospital, Seoul, Republic of Korea) [12], whereas simulated remimazolam *Ce*, estimated according to the Schüttler–Eisenried model, was recorded at 10 s intervals using the Asan Pump software. Both datasets were synchronized to Coordinated Universal Time, allowing each simulated *Ce* value to align temporally with the corresponding BIS and PSI values for subsequent analysis. Table A1 presents the PK parameters and Ke0 implemented in Asan Pump.

The prespecified primary outcome was the paired difference in ABC (BIS−PSI), with a negative value indicating a greater ABC for PSI. The ascending phase was defined as the period from remimazolam infusion initiation to the peak simulated *Ce*, whereas the descending phase represented the subsequent decline in simulated *Ce* after infusion discontinuation. The observation at peak *Ce* was included in both phases.

**Figure A1:**
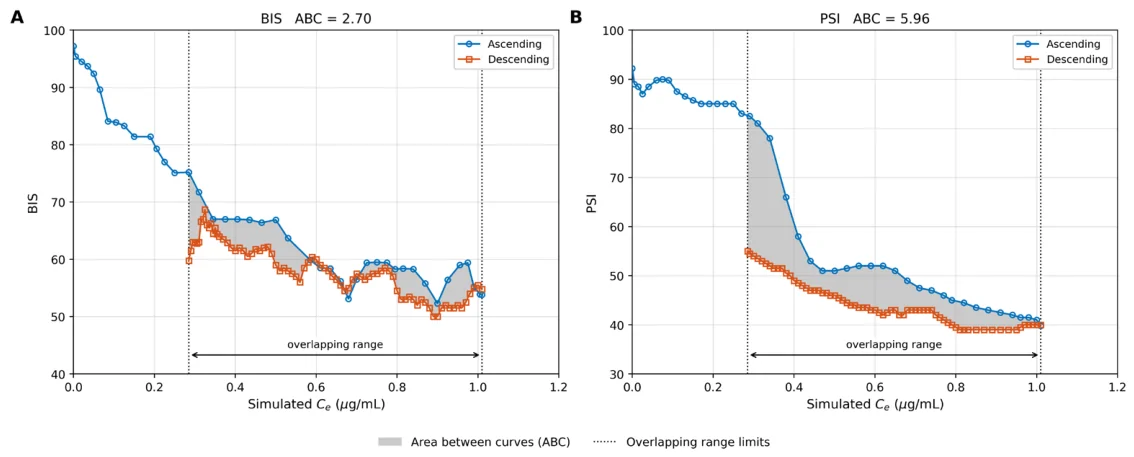
Representative concentration–response loops for (**A**) BIS and (**B**) PSI showing the derivation of ABC. BIS and PSI were plotted against simulated remimazolam *Ce*. Circles represent the ascending phase, while squares represent the descending phase. Dotted vertical lines indicate the concentration range shared by both phases. Within this range, the ascending and descending curves were interpolated, and ABC was calculated by numerically integrating the absolute vertical difference between the curves (index units × µg·mL^−1^). Abbreviations: BIS, bispectral index; PSI, patient state index; ABC, area between curves; *Ce*, effect-site concentration of remimazolam.

The overlapping *Ce* interval for each patient was bounded by the higher minimum (*Ce_min_*) and lower maximum (*Ce_max_*) values of both phases. Within each phase, index values at identical *Ce* values were averaged. Each curve was then linearly interpolated at 500 equally spaced *Ce* points within this interval. ABC was calculated by trapezoidal integration using the following equation:

$$\mathrm{ABC}=\int_{Ce_{\min}}^{Ce_{\max}}\left|f_{\mathrm{asc}}\left(\mathrm{Ce}\right)-f_{\mathrm{des}}\left(\mathrm{Ce}\right)\right|\;d\mathrm{Ce}$$

where f_asc_(*Ce*) and f_des_(*Ce*) represent the interpolated ascending and descending curves, respectively. ABC is expressed in index units × μg/mL^−1^ (Figure A1).

To account for differences in the overlapping *Ce* range and the ranges of the EEG indices, ABC was normalized in a post hoc sensitivity analysis as follows:

$$\mathrm{AB}C_{\mathrm{range}}=\frac{\int_{Ce_{\min}}^{Ce_{\max}}\left|f_{\mathrm{asc}}\left(\mathrm{Ce}\right)-f_{\mathrm{des}}\left(\mathrm{Ce}\right)\right|\;d\mathrm{Ce}}{W\times R}$$

where *W* = *Ce_max_* − *Ce_min_*, and *R* is the range (maximum − minimum) of the interpolated BIS or PSI values across both phases within the overlapping *Ce* interval, calculated separately for each patient. ABC_range_ is dimensionless and ranges (0–1), with larger values indicating greater curve separation relative to the interpolated range of each index.

### 2.4. Study Size

As no previous studies have investigated this topic, this study was designed as exploratory. A target sample size of 20 participants was selected based on the pilot-study sample size guidance summarized in the “Pilot Study Sample Size Rules of Thumb” procedure in PASS 2024 (version 24.0.1; NCSS, Kaysville, UT, USA) [13].

### 2.5. Statistical Methods

Categorical variables are presented as numbers (percentages). Normally distributed continuous variables are presented as mean ± standard deviation, whereas non-normally distributed continuous variables are presented as median (25th–75th percentiles).

For the primary endpoint, ABC measurements obtained from BIS and PSI were compared using a paired analysis because both indices were measured simultaneously in the same patient [14]. The mean paired difference was reported with a two-sided 95% confidence interval (CI) calculated using Student’s t distribution applied to the patient-level paired differences.

As a post hoc sensitivity analysis, the difference in normalized ABC (ABC_range_) was calculated as the normalized BIS ABC minus the normalized PSI ABC and reported as the mean difference with its 95% CI. Additionally, patient-level nonparametric bootstrap procedures were applied to ABC and ABC_range_, with 2000 replicates while maintaining the paired BIS and PSI observations within each patient. For each resampled dataset, the paired median difference was recalculated, and 95% CIs were estimated using the percentile method. The patient-level nonparametric bootstrap analyses of the median paired differences in ABC and ABC_range_ were also repeated with 2000 replicates after excluding the two patients with protocol deviations.

All statistical analyses were performed using MATLAB R2026a (MathWorks, Natick, MA, USA) and SigmaPlot (version 16.0; Grafiti LLC, Palo Alto, CA, USA). Given the exploratory nature of this study, paired differences were presented with 95% CIs.

## 3. Results

### 3.1. Study Patients

Between March and July 2026, 20 patients scheduled for elective laparoscopic cholecystectomy were recruited. No patient was withdrawn or excluded, and all 20 were included in the final analysis. In one patient, remimazolam was administered at 3.0 mg/kg/h instead of the protocol-specified rate of 3.5 mg/kg/h because of an infusion pump setting error. For a second participant, data acquisition ended at 15 min rather than 16 min. Given the small sample size, both patients were included in the data analysis. Table 1 presents patient characteristics, remimazolam dose, apnea incidence, the maximum and minimum simulated remimazolam *Ce* values. No other adverse events were observed.

### 3.2. Primary Outcome

**Figure 2:**
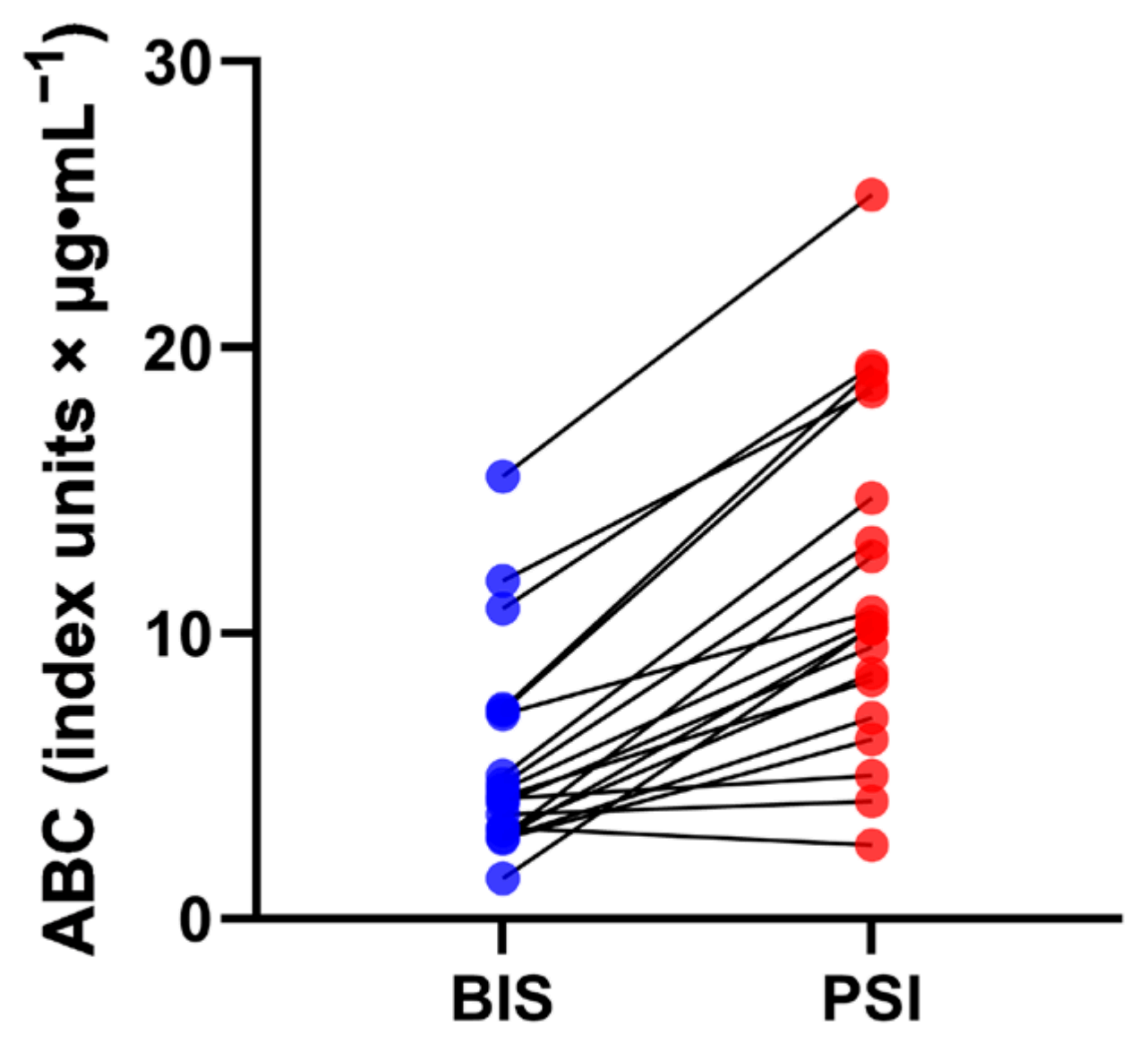
Paired comparison plot of ABC values for BIS and PSI. Lines connect the paired ABC values for each patient. ABC was calculated over the overlapping range of simulated remimazolam *Ce*. Abbreviations: ABC, area between curves; BIS, bispectral index; *Ce*, effect-site concentration; PSI, patient state index.

The mean paired difference in ABC between the two EEG measures was −6.254 (95% CI: −7.945 to −4.563), calculated as BIS minus PSI (Figure 2).

### 3.3. Sensitivity Analysis

**Figure 3:**
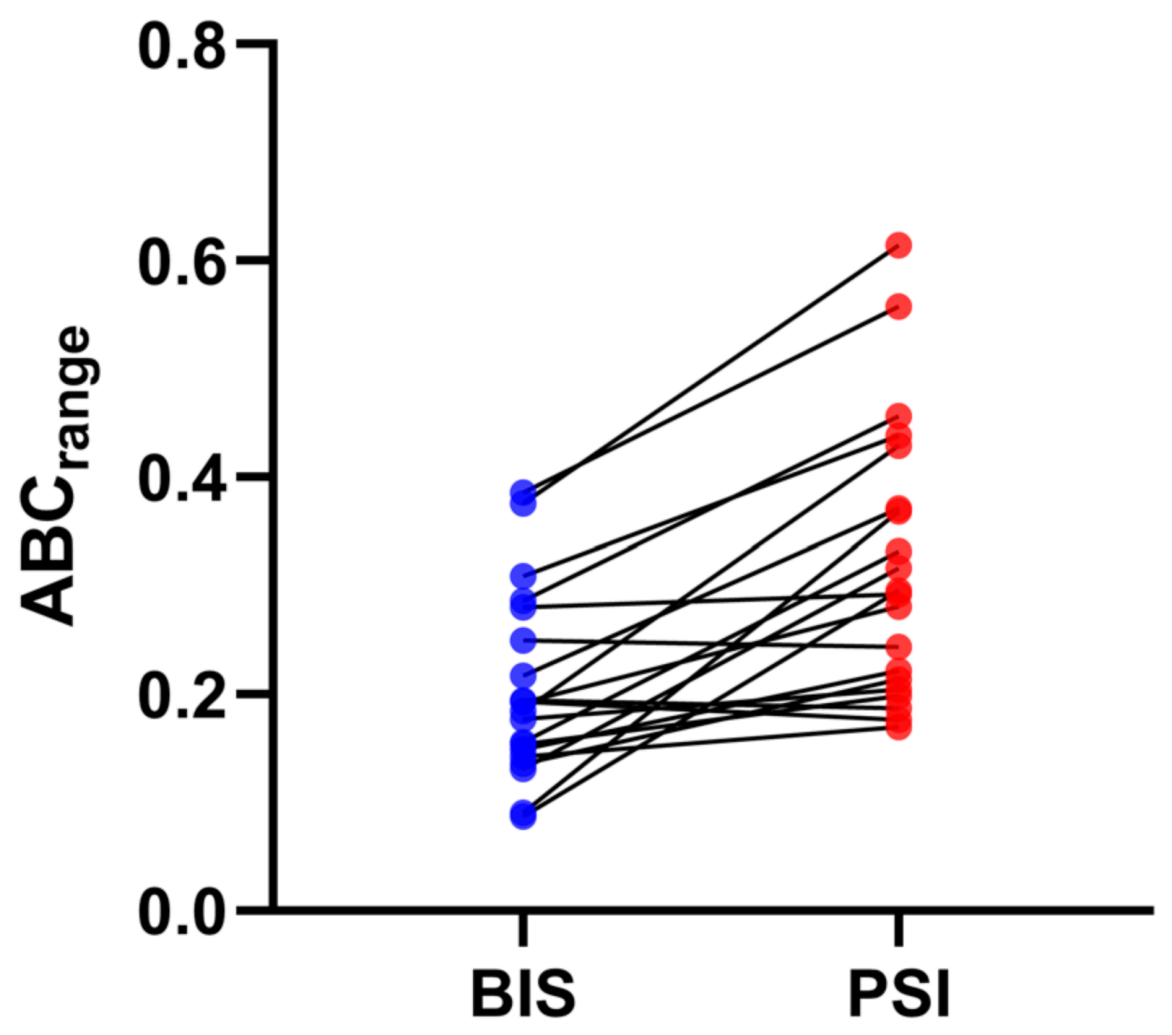
Paired comparison of ABC_range_ values between BIS and PSI. Each line connects paired ABC_range_ values for BIS and PSI from the same patient. Abbreviations: ABC_range_, normalized area between curves; BIS, bispectral index; PSI, patient state index.

The mean difference in ABC_range_ between the two EEG measures, calculated as BIS minus PSI, was −0.114 (95% CI: −0.158 to −0.070; Figure 3).

Table 2 shows the results of the patient-level nonparametric bootstrap analysis of ABC and ABC_range_.

Table 3 summarizes the results of the patient-level nonparametric bootstrap analyses for ABC and ABC_range_ after excluding the two patients with protocol deviations.

## 4. Discussion

The main finding of this prospective, exploratory interventional study was a greater ABC for PSI than for BIS, indicating greater separation between the ascending and descending concentration–response curves for PSI. These findings reflect relationships with model-predicted *Ce* and do not directly demonstrate PD hysteresis. The difference was consistent in the post hoc normalized ABC and patient-level bootstrap analyses. ABC was used to characterize the temporal relationship between each processed EEG index and simulated *Ce*, rather than as a validated measure of clinical sedation depth or recovery. Therefore, a larger ABC does not establish PSI inferiority without an independent reference for hypnotic state.

Effect-site TCI requires PK parameters and the Ke0. Rapid drug bolus administration may impair the accuracy of standard PK models in predicting early arterial or plasma concentrations, potentially resulting in biased Ke0 estimates compared to infusion-based estimates [15]. In this study, remimazolam was administered through continuous infusion, consistent with the regimen used to develop the Schüttler–Eisenried model. The *Ce* associated with a 50% probability of loss of consciousness is approximately 0.6 µg/mL [4,9]. Therefore, a fixed infusion regimen was used to increase the simulated *Ce* above 0.9 µg/mL, corresponding to a 100% probability of loss of consciousness [9]. The mean simulated *Ce* decreased to 0.37 µg/mL during the observation period. A *Ce* of 0.1 µg/mL indicates a 100% probability of regaining responsiveness [9]. In the simulation, *Ce* was predicted to fall below 0.1 µg/mL approximately 35 min after infusion ended, a time point not feasibly observed in the operating room. Therefore, the descending phase evaluated in this study did not encompass the full concentration range associated with recovery.

To our knowledge, this is the first report to examine the ascending and descending slopes of simulated remimazolam *Ce* relative to concurrent BIS and PSI changes. Remimazolam TCI was used with an Asan Pump based on the Schüttler–Eisenried model in a previous study, reporting identical prediction probabilities of 0.76 for PSI and BIS relative to *Ce* [8]. The prediction probabilities for MOAA/S versus PSI and MOAA/S versus BIS were also similar (0.81 and 0.80, respectively) [8]. However, the study assessed only the ascending phase of *Ce*, rather than the descending phase. A study comparing BIS and PSI during remimazolam-based general anesthesia reported a strong overall correlation, with differences during induction and emergence [16]. The present study assessed both indices during remimazolam infusion and after infusion ended without concurrent hypnotic or opioid administration during the observation period. The present study design enabled comparison of the two processed EEG indices during relatively isolated changes in remimazolam exposure. Nevertheless, *Ce* was simulated rather than measured, and the Schüttler–Eisenried model was developed in healthy men aged 18–40 years with ASA-PS I [4]. Demographic and physiological differences between the model-development population and this cohort, which included women and patients with ASA-PS II, may have reduced the accuracy of the simulated *Ce*.

Studies report inconsistent relationships between clinical responsiveness and processed EEG indices during remimazolam administration. During remimazolam TCI, MOAA/S correlated similarly with BIS and PSI (*r* = 0.84 and 0.82, respectively) [8]. In contrast, after a single remimazolam bolus, MOAA/S correlated less strongly with PSI than with BIS (*r* = 0.390 and 0.568, respectively) [17]. PSI also declined more slowly than BIS; however, temporal responses were not formally compared [17]. These findings suggest that BIS and PSI may diverge more during rapid changes in drug concentration.

The larger ABC for PSI may reflect differences in signal processing and temporal response between the two monitors. BIS updates every second, whereas PSI updates every 2 s [18]; however, the effect of update frequency on ABC could not be determined in this study. Differences in BIS and PSI sensitivity to remimazolam-related EEG changes may also have contributed. Given that raw EEG data and device-specific processing delays were not assessed, this study could not distinguish signal-processing and temporal response effects from true pharmacodynamic differences.

A major strength of this study is the simulation of the ascending and descending *Ce* phases without concurrent administration of other anesthetic agents. Although BIS and PSI both use a 0–100 scale, their target ranges for anesthetic depth differ. Normalization accounts for the overlapping *Ce* range and the observed within-patient range of each index, facilitating comparison of relative curve separation. In the post hoc analysis, PSI showed greater normalized curve separation than BIS. This finding is consistent with the unnormalized ABC results but does not establish BIS monitor superiority.

This study has some limitations. First, loss and recovery of consciousness were not assessed using the MOAA/S scale because external stimuli can change the processed EEG measures [19]. Consequently, while the effects of external stimulation on BIS and PSI were minimized, the relationship between the observed EEG responses and clinical sedation level could not be evaluated. Second, *Ce* was model-predicted rather than measured against remimazolam concentrations, and the findings partly depend on the PK/PD model used. Third, the observation period ended before simulated *Ce* reached the level associated with recovery of responsiveness. Thus, the descending phase was evaluated over a limited concentration range, precluding conclusions regarding recovery of responsiveness. Fourth, minor protocol deviations occurred in two patients: one received a lower remimazolam infusion rate, and the other had a shorter recording period. Finally, this was a small, single-center exploratory study of 20 patients. Larger studies are needed to confirm these findings.

## 5. Conclusions

In this prospective exploratory interventional study, PSI showed greater separation between the ascending and descending concentration–response curves than BIS during changes in simulated remimazolam *Ce*. These findings suggest that BIS and PSI may differ in their temporal responses to changes in remimazolam concentrations but do not establish BIS superiority for assessing hypnotic state. Given the small sample size and use of model-predicted rather than measured concentrations, these findings remain exploratory and require confirmation in larger studies incorporating measured plasma remimazolam concentrations and validated clinical sedation assessments.

## Figures and Tables

**Table 1:** Baseline characteristics, apnea incidence, remimazolam dose, and simulated *Ce* during the observation period (*N* = 20).

Variable	Values
Age (yr)	53.0 (42.0, 58.8)
Female	6 (30%)
Height (cm)	167.1 ± 6.8
Weight (kg)	71.8 ± 10.9
ASA-PS I	8 (40%)
ASA-PS II	12 (60%)
Apnea ^a^	4 (20%)
Highest simulated *Ce* ^b^ (µg/mL)	1.15 ± 0.16
Lowest simulated *Ce* ^c^ (µg/mL)	0.37 ± 0.08
Remimazolam dose ^b^ (mg/kg)	0.32 ± 0.01

Values are presented as numbers (%), mean ± standard deviation, or median (25th–75th percentile). ^a^ defined as the absence of an end-tidal CO_2_ waveform for >10 s. ^b^ During the observation period. ^c^ At the end of the observation period. Abbreviations: ASA-PS, American Society of Anesthesiologists physical status; *Ce*, effect-site concentration.

**Table 2:** Patient-level nonparametric bootstrap estimates of the paired difference in ABC and ABC_range_ between BIS and PSI (*N* = 20).

Measure	Median Paired Difference ^a^ (95% CI)
ABC	−6.241 (−8.752 to −4.133)
ABC_range_	−0.109 (−0.174 to −0.036)

CIs were calculated using patient-level nonparametric bootstrap resampling with 2000 replicates. ^a^ Calculated as BIS minus PSI; negative values indicate a larger ABC or ABC_range_ value for PSI. Abbreviations: ABC, area between curves; ABC_range_, normalized area between curves; BIS, bispectral index; CI, confidence interval; PSI, patient state index.

**Table 3:** Patient-level nonparametric bootstrap estimates of the paired differences in ABC and ABC_range_ between BIS and PSI after excluding two patients with protocol deviations (*N* = 18).

Measure	Median Paired Difference ^a^ (95% CI)
ABC	−6.241 (−9.102 to −4.133)
ABC_range_	−0.109 (−0.173 to −0.045)

CIs were calculated using patient-level nonparametric bootstrap resampling with 2000 replicates. ^a^ Calculated as BIS minus PSI; negative values indicate a larger ABC or ABC_range_ value for PSI. Abbreviations: ABC, area between curves; ABC_range_, normalized area between curves; BIS, bispectral index; CI, confidence interval; PSI, patient state index.

## Data Availability

The original data presented in the study and MATLAB code (ABC, ABC_range_, sensitivity analysis) are openly available in the Zenodo repository at https://doi.org/10.5281/zenodo.23117103.

## Abbreviations

The following abbreviations are used in this manuscript:

TCI	Target-controlled infusion
Ce	Effect-site concentration
PK	Pharmacokinetic
PD	Pharmacodynamic
PSI	Patient state index
BIS	Bispectral index
Ke0	Plasma-to-effect-site equilibration rate constant
MOAA/S	Modified Observer’s Assessment of Alertness and Sedation
EEG	Electroencephalogram
ASA-PS	American Society of Anesthesiologists physical status
ABC	Area between curves
CI	Confidence interval

## Appendix A

**Table A1:** Remimazolam pharmacokinetic parameters and Ke0 implemented in Asan Pump. Parameters were based on the Schüttler–Eisenried model [4].

Parameter	Value
V1 (L)	0.062667 × kg
V2 (L)	14.5
V3 (L)	15.5
CL (L/min)	1.14
Q2 (L/min)	1.04
Q3 (L/min)	0.19
Ke0 (/min)	0.27

The Ke0 of 0.27 min^−1^ was derived using MOAA/S as the pharmacodynamic endpoint. Body weight was expressed in kilograms. Abbreviations: V1, central compartment volume; V2, rapid peripheral compartment volume; V3, slow peripheral compartment volume; CL, elimination clearance; Q2 and Q3, intercompartmental clearances between the central and respective peripheral compartments; Ke0, plasma-to-effect-site equilibration rate constant.
